# Histatin 1 enhanced the speed and quality of wound healing through regulating the behaviour of fibroblast

**DOI:** 10.1111/cpr.13087

**Published:** 2021-07-13

**Authors:** Liuhanghang Cheng, Xiaoxuan Lei, Zengjun Yang, Yanan Kong, Pengcheng Xu, Shiya Peng, Jue Wang, Cheng Chen, Yunqing Dong, Xiaohong Hu, Xiaorong Zhang, Tymour Forouzanfar, Gang Wu, Xiaobing Fu

**Affiliations:** ^1^ Research Center for Tissue Repair and Regeneration Affiliated to the Medical Innovation Research Department and 4th Medical Center PLA General Hospital and PLA Medical College Beijing China; ^2^ PLA Key Laboratory of Tissue Repair and Regenerative Medicine and Beijing Key Research Laboratory of Skin Injury, Repair and Regeneration Beijing China; ^3^ Research Unit of Trauma Care, Tissue Repair and Regeneration Chinese Academy of Medical Sciences Beijing China; ^4^ Department of Oral and Maxillofacial Surgery/Pathology Amsterdam UMC and Academic Center for Dentistry Amsterdam (ACTA) Vrije University Amsterdam (VU) Amsterdam Movement Science Amsterdam The Netherlands; ^5^ Department of Burn and Plastic Surgery General Hospital of Southern Theater Command Guangzhou China; ^6^ Department of Dermatology Southwest Hospital Third Military Medical University (Army Medical University) Chongqing China; ^7^ Department of Plastic Surgery First Affiliated Hospital of Anhui Medical University Hefei China; ^8^ Department of Dermatology and Rheumatology Immunology Xinqiao Hospital Third Military Medical University (Army Medical University) Chongqing China; ^9^ State Key Laboratory of Trauma, Burns, and Combined Injury Institute of Burn Research The First Affiliated Hospital of Army Medical University (the Third Military Medical University) Chongqing China; ^10^ Chongqing Key Laboratory for Disease Proteomics Chongqing China; ^11^ The First School of Clinical Medicine Southern Medical University Guangzhou China; ^12^ Department of Oral Implantology and Prosthetic Dentistry Academic Center for Dentistry Amsterdam (ACTA) University of Amsterdam (UvA) and Vrije Universiteit Amsterdam (VU) Amsterdam The Netherlands

**Keywords:** collagen deposition, fibroblast, Histatin 1, mechanical properties, wound healing

## Abstract

**Objectives:**

Histatin 1(Hst 1) has been proved to promote wound healing. However, there was no specific study on the regulation made by Hst 1 of fibroblasts in the process of wound healing. This research comprehensively studied the regulation of Hst 1 on the function of fibroblasts in the process of wound healing and preliminary mechanism about it.

**Materials and methods:**

The full‐thickness skin wound model was made on the back of C57/BL6 mice. The wound healing, collagen deposition and fibroblast distribution were detected on days 3, 5 and 7 after injury. Fibroblast was cultured in vitro and stimulated with Hst 1, and then, their biological characteristics and functions were detected.

**Results:**

Histatin 1 can effectively promote wound healing, improve collagen deposition during and after healing and increase the number and function of fibroblasts. After healing, the mechanical properties of the skin also improved. In vitro, the migration ability of fibroblasts stimulated by Hst 1 was significantly improved, and the fibroblasts transformed more into myofibroblasts, which improved the function of contraction and collagen secretion. In fibroblasts, mTOR signalling pathway can be activated by Hst 1.

**Conclusions:**

Histatin 1 can accelerate wound healing and improve the mechanical properties of healed skin by promoting the function of fibroblasts. The intermolecular mechanisms need to be further studied, and this study provides a direction about mTOR signalling pathway.

## Graphical abstract

Histatin 1 may be activated by G protein coupled receptors on the surface of cell membrane. Subsequently, PI3K / Akt / mTOR signaling pathway was activated to promote the migration of fibroblasts and the transformation of fibroblasts into myofibroblasts. By regulating the function of fibroblasts, it can improve the healing speed of acute wound and the mechanical properties of skin after healing.

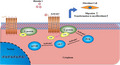

## 1 INTRODUCTION

Acute wound of skin and soft tissue is one of the most popular disease in clinical work, and it is usually caused by burning, mechanical force, physical or chemical stimulation. Wound healing not only influences the appearance of the patients, but also has a great impact of the function. Although there are some targeted methods for wound healing, it does not fundamentally solve the problem.^1^, ^2^ Wound healing consists of several distinct but overlapping stages, including haemostasis, inflammation, tissue remodelling and maturation.^3^, ^4^, ^5^ Fibroblast, which play a significant role in wound healing,^6^ could transform into myofibroblast expressing alpha‐smooth muscle actin (α‐SMA) by the influence of transforming growth factor‐β1 (TGF‐β1) after wound formation.^7^ TGF‐β1 regulates functions of fibroblasts during wound healing and is produced by macrophages and many other cells.^8^ In the early stage of wound healing, fibroblasts and myofibroblasts secret intercellular matrix that constitutes an important part of granulation tissue and accelerate the wound closure through the contraction of the wound.^9^, ^10^ The speed of wound closure and filling determines the prognosis of wound greatly.^11^ In addition, in the late stage of wound healing, myofibroblasts determine the mechanical properties of the skin by affecting the deposition and distribution of collagen and other fibres.^12^

Histatin is a family of histidine rich polypeptides that exist in saliva.^13^ They were paid attention to for their antifungal effects at first. However, in recent years, the activation of tissue polypeptides on cells has gradually become a hotspot. Among them, Hst 1 most widely studied in promoting wound healing.^14^ In oral cavity, Hst 1 can accelerate wound healing by haemostasis, promoting angiogenesis, epithelial cell migration and adhesion to reconstruct epithelial barrier.^15^ In addition to oral epithelial cells, Hst 1 can activate many other cells.^16^, ^17^ Recent studies have also shown that Hst 1 can inhibit the expression of interleukin‐1β (IL‐1β), C‐reactive protein (CRP) and CD68 in wound healing.^18^ However, there is no study about the mechanical properties of the healed skin after treatment with Hst 1.

Previous studies have shown that Hst 1 can promote the migration of fibroblasts,^19^ but there is no detailed study on its other functions. Hst 1 can significantly reduce the wound area in the process of wound healing. Previous studies suggested that this was mainly related to its promotion of epithelial cell migration,^20^ while ignoring the contraction effect of myofibroblasts. Although some experiments tested the effect of Hst 1 on collagen deposition,^18^, ^21^ they did not study the mechanical properties of the skin after wound healing or proved the effect of fibroblasts in vitro. In this study, the effects of Hst 1 on the characteristics and function of fibroblasts were studied completely in vivo and in vitro, and we tried to explore the mechanism of the effect.

## 2 METHODS AND MATERIALS

### 2.1 Hst 1 preparation

The Hst 1(≥95% purity) was synthesized by manufacturer (SynPeptide Co., Ltd., Nanjing, China). Hst 1 was dissolved in 0.9% NaCl solution into a concentration of 1 mmol/L and stored at −20°C as a solid. Hst 1 was diluted to a working concentration of 10 μmol/L during the experiment.

### 2.2 Experimental animals and wound model

Thirty‐eight clean grade C57/BL6 male mice (Experimental Animal Center of the Army Medical University), which were 6‐8 weeks old and 25‐30 g weight, were kept at 18‐25°C and 50% constant humidity (Animal Center of Southwest Hospital of Chongqing). All the mice were allowed to access normal food and water freely. Five mice were kept in one cage before making the wound model, and one mouse was kept per cage while the model was completed.

The hair on the back of the mice were shaved carefully and sterilized with lodophor. The mice were anaesthetized by intraperitoneal injection of 1% pentobarbital solution (Sigma, USA) via intraperitoneal injection for 5 mL/kg. After anaesthesia, two full‐thickness skin wounds with a diameter of 1 cm were made on both sides of the back of the mice by a puncture biopsy instrument. The experimental mice were treated with 200 μL 10 μmol/L Hst 1 solution every day, while the control group were treated with equal volume deionized water. All the mice were photographed every other day and randomly sacrificed on the 3, 5, 7, 11 day and 10 days after healing to detected the wound skin.

### 2.3 Wound closure analysis and histological analysis

Photographs were taken every other day after the surgery, and the area of wound was calculated by the image analysis software (Image J, Rawak Software, Inc Germany). *W*_0_ was defined as the initial area of the wound, and *W*_t_ was defined as the residual wound area. The wound healing rate (% area of wound healed) was measured as follows:

$$W_{\%}\left(\%\text{of closed wound area}\right)=\left(W_{0}‐W_{t}\right)/W_{0}\times 100\%$$

Skin samples were taken at 3, 5, 7, 11 after surgery and 1 week after healing. All the specimens were fixed in 4% paraformaldehyde, dehydrated, paraffin‐embedded and made into 5 μm sections. Haematoxylin (Beyotime, China) and eosin (H&E) staining, Masson staining, reticular fibre staining and Victoria blue staining were performed for the sections, and all sections were observed and photographed under the microscope. ImageJ software was used to measure the length of wound healing and granulation tissue area. The length of wound healing represents the ability of re‐epithelialization. ImageJ was also used to analyse the optical density of various fibres staining and calculate the fibre composition after wound healing.

### 2.4 Immunohistochemistry and immunofluorescence staining

Paraffin sections were dewaxed and hydrated with xylene and gradient alcohol. After that, the sections were heated for antigen retrieval. The endogenous peroxidase was inactivated with 3% H_2_O_2_ solution after cooling to room temperature (only immunohistochemistry). Ten percent normal goat serum was used for 30 minutes at room temperature to block the antigen, and the primary antibody was incubated overnight at 4°C. Biotinylated secondary antibody (Zhongshan Biology Co. Ltd, China) was incubated at room temperature for 30 minutes. In the immunohistochemical staining, diaminobenzidine was used for colour development and haematoxylin (Beyotime, China) was used for nuclear staining. Results were taken by a microscope (Olympus, Japan). The positive results were quantified by ImageJ software, and at least three sections were randomly selected for each group. Five different high‐power field of view were randomly selected from each slice for calculation and statistics.

The primary antibody used is as follows: anti‐α‐smooth muscle actin (α‐SMA) (1:150, Abcam, UK); anti‐transformation growth factor‐β (TGF‐β) (1:200, Abcam, UK); anti‐vamentin (1:200, Abcam, UK).

### 2.5 Cell culture and biological function of fibroblast

Fibroblast (NIH/3T3; GNM 6; Chinese Academy of Sciences, China) was cultured in a 5% CO_2_ incubator at 37°C. The cells were passaged every 3 days, and 3.75 × 10^5^ cells were inoculated into each 25 cm^2^ culture bottle. The control group was cultured by completed DMEM medium, while the medium of experimental group was added 10 μmol/L Hst 1.

#### 2.5.1 Cell migration

Fibroblasts were planted into 6‐well plates with the number of 2 × 10^6^/well and cultured to 80% density. Cell were starved for 8‐12 hours, and mitomycin C (Sigma Aldrich, USA) was added into medium by 15 μg/ml. After culturing in a 5% CO_2_ incubator at 37°C for 3 hours, the medium was replaced with normal completed medium. The scratch was made by the 200 μL pipette tip on the bottom of the well vertically with the same force and washed by PBS twice. Photographs were taken every 12 hours for 48 hours by a microscope, and the residual area of scratch was calculated by Image J and the initial area was the area at 0 hour.

#### 2.5.2 Cell proliferation

According to the kit instruction of Edu (Invitrogen, USA), cells were incubated with 5 μmol/L/well of Edu for more than 3 hours. After treatment of cells in each group, the cells were cultured in a 5% CO_2_ incubator at 37°C for 3 hours and carried out treatment according to the instruction. Finally, the cells were tested and analysed with a flow cytometer (Attune, Applied Biosystems AB, USA).

#### 2.5.3 Cell viability

Cell viability was determined by the CCK8 method. Cell suspension was inoculated in 96 well plate with 2000 cells each well (100 μL/well). The cells were pre‐incubated in the incubator for 12 hours to make the cells attachment and added influencing factors according to the experimental group. After incubation for 0, 12, 24, 48 and 72 hours, 10 μL CCK8 detection solution was added into each well, and OD value was detected by microplate reader.

#### 2.5.4 Cell apoptosis

3T3 cells were cultured with stimulation according to the experimental groups for 24 hours and collected by centrifugation (290 *g*/5 min). Cells were washed in PBS and then resuspended in 200 μL binding buffer (1×) with density of 2‐5 × 10^5^/mL. Then, the cells were treated according to the instructions of the Invitrogen Annexin V‐FITC apoptosis detection kit (Thermo Fisher Scientific, USA). Flow cytometry was used for data analysis.

### 2.6 Cell contraction test

0.1 mol/L NaOH solution was used to adjust the pH of commercial rat tail type I collagen (Solarbio, China) to neutral. 1 × 10^5^ 3T3 cells were resuspended in the culture medium and mixed with pH neutral rat tail collagen solution to make the concentration of collagen 1 mg/mL. 1 mL of rat tail collagen solution containing cells was added into each well of 12 well plate, and the plate was placed at room temperature for 20 minutes to make collagen coagulate into gel. After adding suitable volume medium culture and different stimulant for each group, the plate was cultured in a 5% CO_2_ incubator at 37°C. Photographs were taken every 12 hours for each well, and the residual area of collagen was calculated by image J.

### 2.7 ELISA and Western blot

The supernatant was extracted from cell culture medium after 3 days of culture under different conditions. The concentrations of type I collagen were determined by mouse type I collagen quantification ELISA kit (Elabscience Biotechnology Co., Ltd, China). For immunoblot, harvested cells were lysed on ice using RIPA lysis buffer (Beyotime, China) with protease and phosphatase inhibitors for 30 minutes. Then, the lysates were centrifuged for 20 minutes at 4°C, 13523 *g*. Supernatant was transferred to another tube and quantitated using BCA protein assay kit (Thermo Fisher Scientific, USA). Loading buffer (Beyotime, China) was added to supernatant, and samples were denatured at 100°C for 10 minutes. Total protein extracts were subjected to SDS‐polyacrylamide gel electrophoresis, transferred to PVDF membrane, blocked with 5%BSA in TBST. Primary antibodies were incubated at 4°C overnight, and secondary antibodies were incubated in room temperature for 1 hour. Finally, signals were detected and images were taken.

The primary antibody we used are as follows: anti‐α‐smooth muscle actin (α‐SMA) (1:1000, Abcam, UK); anti‐collagen I (1:1000, Abcam, UK); anti‐collagen III (1:1000, Abcam, UK); anti‐Akt (1:1000, CST, USA); anti‐pAkt (1:1000, CST, USA); anti‐PI3K (1:1000, CST, USA); anti‐pPI3K (1:1000, CST, USA); anti‐mTOR (1:1000, CST, USA); anti‐pmTOR (1:1000, CST, USA).

### 2.8 Tensile failure and stress relaxation

Before the experiment, we used vernier caliper to measure the specification (length, width, thickness) of each skin sample and the samples were fixed on the sensor of the Instron 5567 materials testing system (Instron, USA) and the force measurement was zeroed. After adjusting the upper and lower clamps on the same vertical horizontal plane to ensure that the stress direction of the skin is vertical, we tightened the upper and lower clamps to prevent the sample from sliding out during the experiment. For tensile failure test, when the test began, the sample was elongated from 0 to failure with constant rate (50 mm/min). All data were recorded by a computer software. For stress relaxation test, the sample was slowly stretched to the target shape variable (20%) at a constant speed (50 mm/min) and maintained for 120 seconds. Three different samples were taken from each group, and PBS was used to keep the samples moist during the test.

### 2.9 Quantitative real‐time PCR (qPCR)

qPCR assay was used to detect the expression of related indicators at mRNA level. TRIzol reagent (Ambion Life Technologies, USA) was used to isolate total RNA from the cells. After calculating the concentration of RNA, a cDNA synthesis kit (Takara, Japan) was used for reverse transcription. The prepared cDNA template and PCR kit were used to add samples according to the instructions, and then, a instrument (CFX Connect™, BIO‐RAD, USA) was used for qPCR reaction. Primers for α‐SMA were designed based on the statistics from NCBI and Pubmed. GAPDH was used as the internal reference.

α‐SMA F:CCC AGA CAT CAG GGA GTA ATG G

α‐SMA R:TCT ATC GGA TAC TTC AGC GTC A

GAPDH F:GGT TGT CTC CTG CGA CTT CA

GAPDH R:TGG TCC AGG GTT TCT TAC TCC

### 2.10 Ethics approval

Animal experiments were approved by the Medical and Ethics Committee of Southwest Hospital, Third Military Medical University (Army Medical University), Chongqing, China.

### 2.11 Statistical analysis

All results were presented as the mean ± SD. Statistical analysis was performed by GraphPad Prism 5.0. An independent sample *t* test was used for comparison between two groups at the same time point, and two‐way ANOVA was used for comparison between two groups at multiple time points. A value of *P* < .05 was considered statistically significant. ∗*P* < .05, ∗∗*P* < .01.

## 3 RESULTS

### 3.1 Hst 1 promoted skin wound construction

**FIGURE 1:**
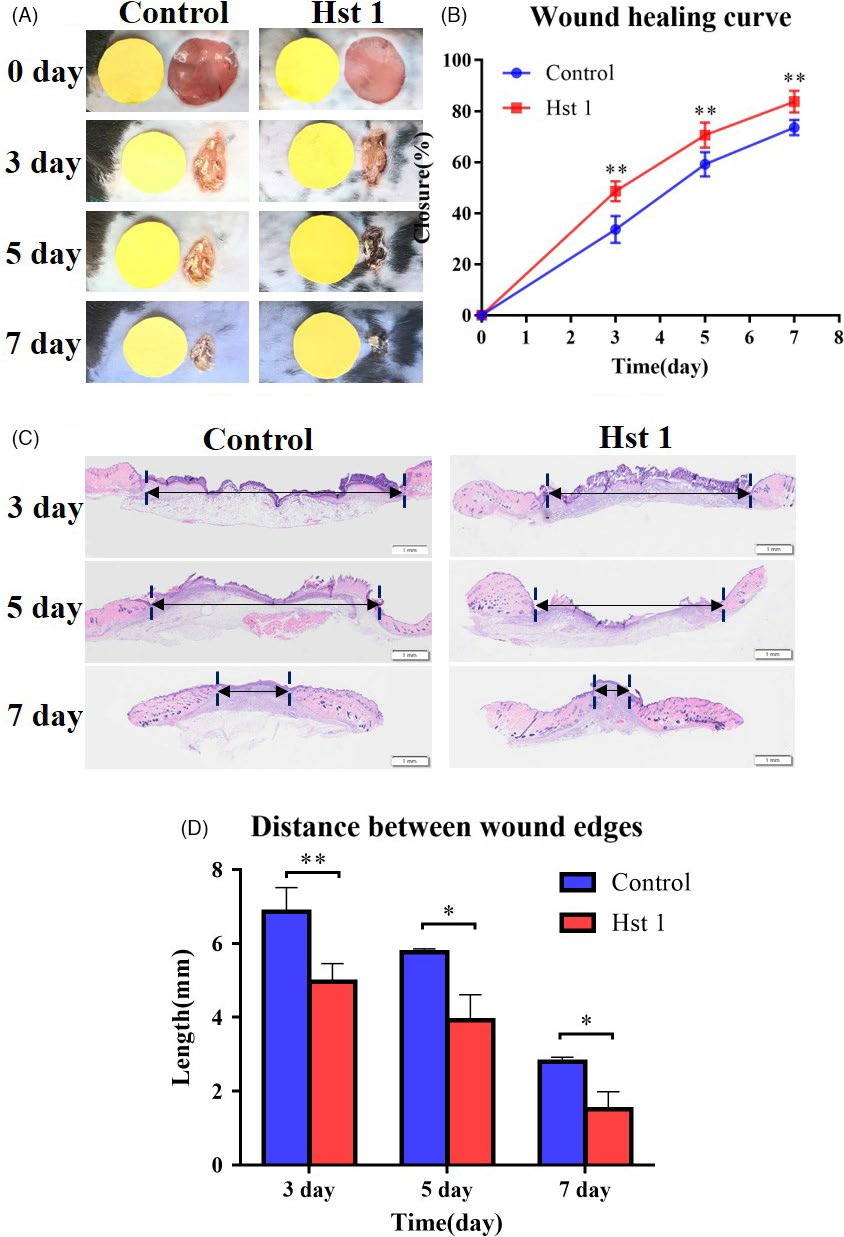
Overall situation of wound healing in vivo in C57 mouse. A, Round wounds with a diameter of 1.0 cm were made on both sides of the mouse body. Hst 1(10 μmol/L) solution was added on the wound surface of experiment group and equal volume deionized water for the control group. Each mouse was photographed every other day. B, Healing rate of wound in mouse. The area of wound healed in every mouse and the ratio to the initial total area were calculated by an image analyser. Vertical axis, healing wound area expressed as % area; horizontal axis, time. C, The results of HE staining of the wound skin at 3, 5 and 7 d. D, Length between wound edge was calculated by an image analyser (At last 3 sections were chosen at each time for each group). Vertical axis, length of wound edge; horizontal axis, time. Data are shown as mean ± SE. (*P < .05; **P < .01)

In order to study the effect of Hst 1 on skin wound healing, a full‐thickness skin wound model was made in C57 mice. The wound healing progress was analysed at different time points. The results showed that the wound healing rate of the experimental group was significantly faster than control group on the 3, 5 and 7 days after injury (Figure 1A,B). The results of HE staining were consistent with the wound area and wound healing rate (Figure 1C,D).

### 3.2 Hst 1 could promote the deposition of wound stroma and granulation filling in vivo

**FIGURE 2:**
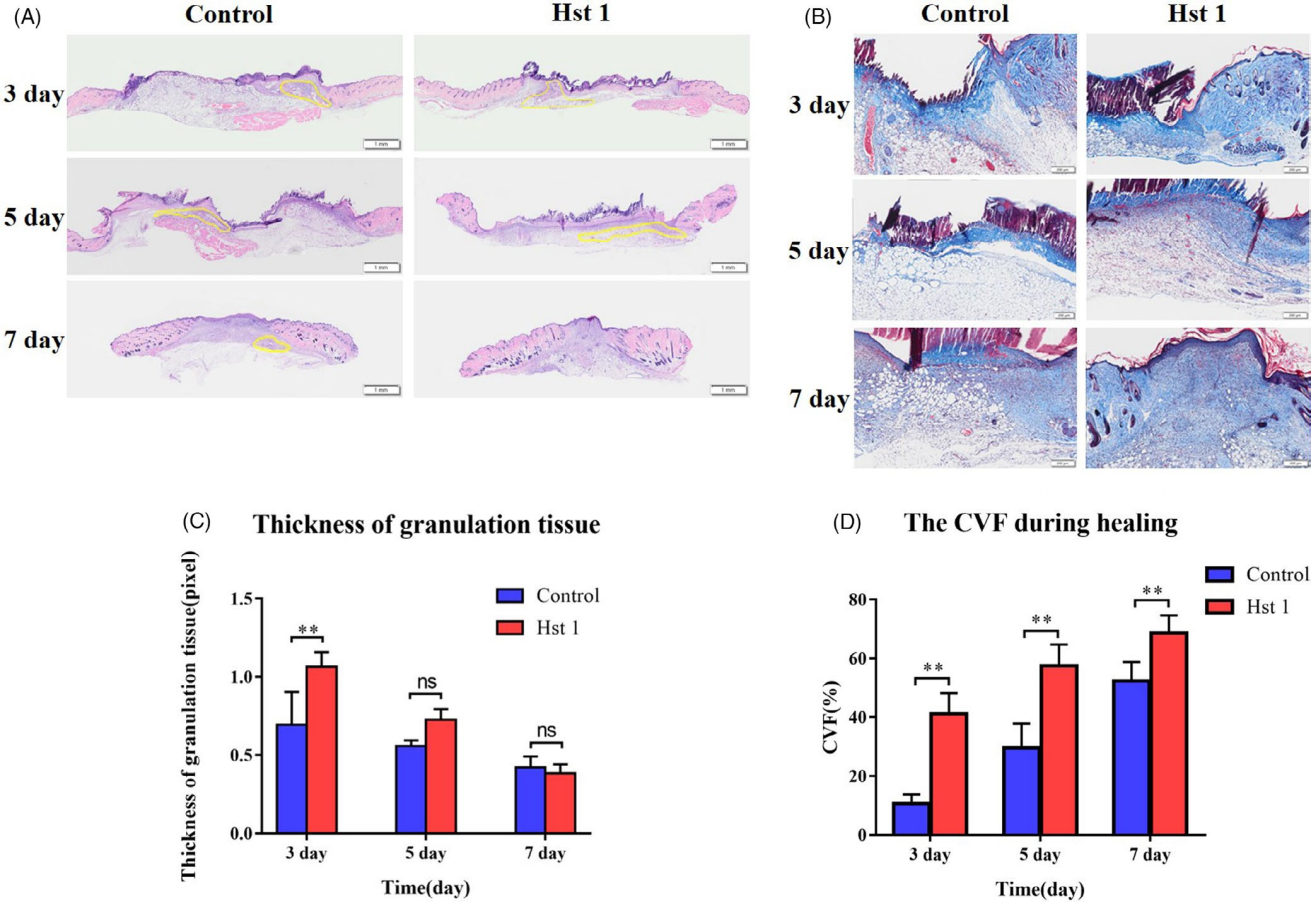
Distribution of granulation tissue and extracellular matrix during healing. A, The HE staining showed the thickness of granulation tissue. B, Statistical data of granulation tissue area during healing process. Staining of interstitial components in granulation tissue. C, Masson staining was used to stain the collagen fibres in the skin sections during the healing process. D, Collagen volume fraction (CVF) during healing process, Vertical axis, CVF; horizontal axis, time. Data are shown as mean ± SE. (*P < .05; **P < .01)

The area of granulation tissue in Hst 1 treatment group was also higher than control group at the same time point (Figure 2A,B). Meanwhile, Masson staining results showed that the content of collagen fibres in the wound treated with Hst 1 was higher than control group on the 3, 5 and 7 day after injury (Figure 2C,D).

### 3.3 Hst 1 promoted fibroblast migration and its transformation of myofibroblast in vivo

**FIGURE 3:**
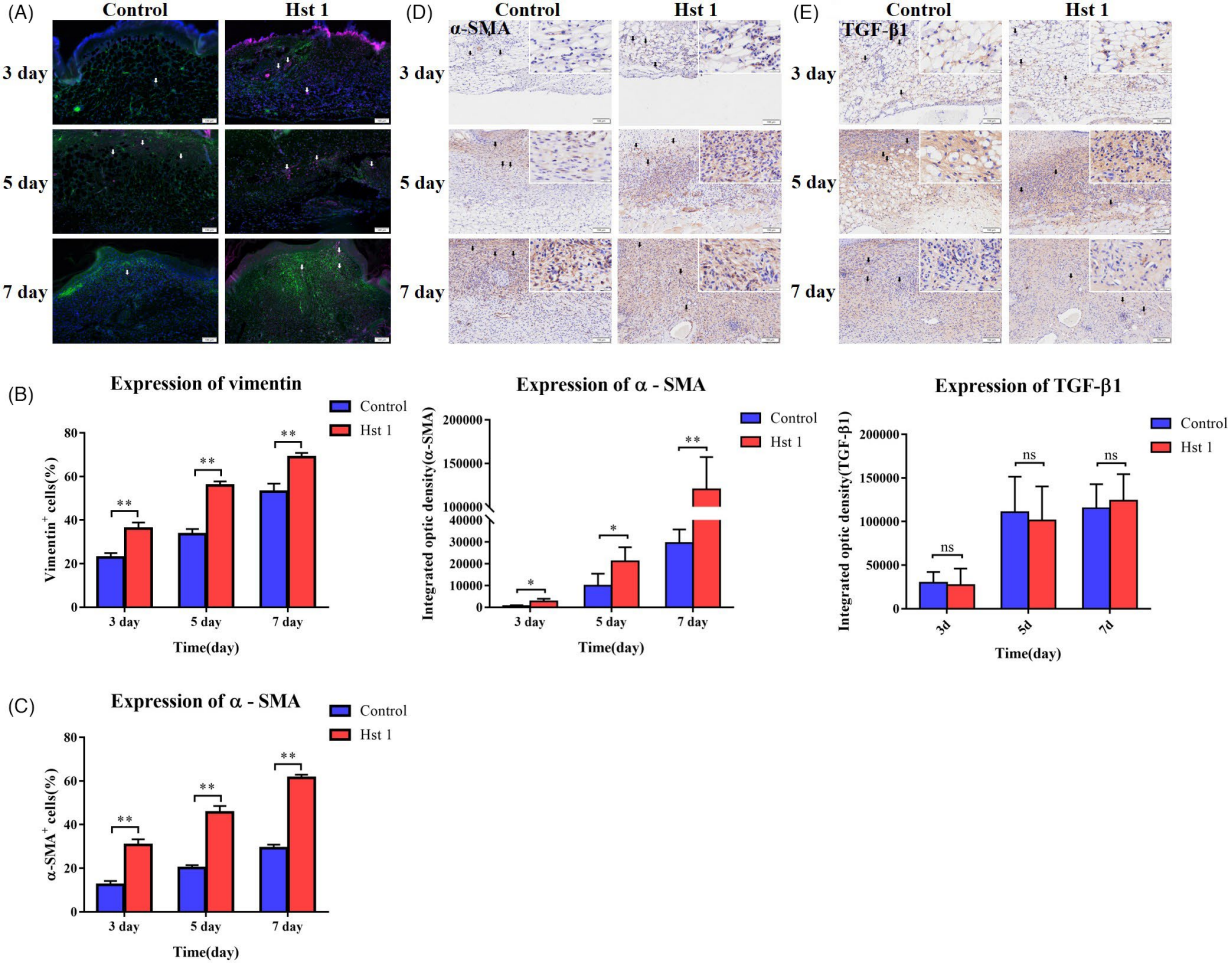
Distribution of fibroblasts and related markers in wounds. A, Distribution of fibroblasts and myofibroblasts in wound. The wound sections were stained with immunofluorescence. DAPI was labelled blue fluorescence, vimentin was green and α‐SMA was purple. B, Percentage of fibroblasts in the wound, vertical axis, number of vimentin^+^ cells; horizontal axis, time. C, Percentage of myofibroblasts in the wound, vertical axis, number of α‐SMA^+^ cells; horizontal axis, time. D, Immunohistochemical staining of α‐SMA in wound sections and the optical density of each section was calculated by image analyser. Vertical axis, value of optical density; horizontal axis, time. E, Immunohistochemical staining of TGF‐β1 in wound sections. The optical density of each section was calculated by image analyser. Vertical axis, value of optical density; horizontal axis, time. Data are shown as mean ± SE. (*P < .05; **P < .01)

Immunofluorescence staining showed the distribution of fibroblasts and myofibroblasts in the wound. The cells labelled with green fluorescence (vimentin) represented fibroblasts, while the cells labelled with green and purple fluorescence (α‐SMA) represented myofibroblasts. The number of fibroblasts and the transformation rate of myofibroblast in the wound treated with Hst 1 were higher than control group (Figure 3A‐C). As an important marker of myofibroblasts, the expression of α‐SMA in Hst 1 treated group was higher than control group (Figure 3D). The distribution of fibroblasts and myofibroblasts was shown by immunohistochemical and immunofluorescence staining. There was no obvious difference of the expression of TGF‐β1, which is a significant factor to promote the transformation of fibroblasts into myofibroblasts, between the control group and the Hst 1 treated group (Figure 3E).

### 3.4 Hst 1 improved the mechanical properties of wound healing skin through regulating the fibre deposition and arrangement

**FIGURE 4:**
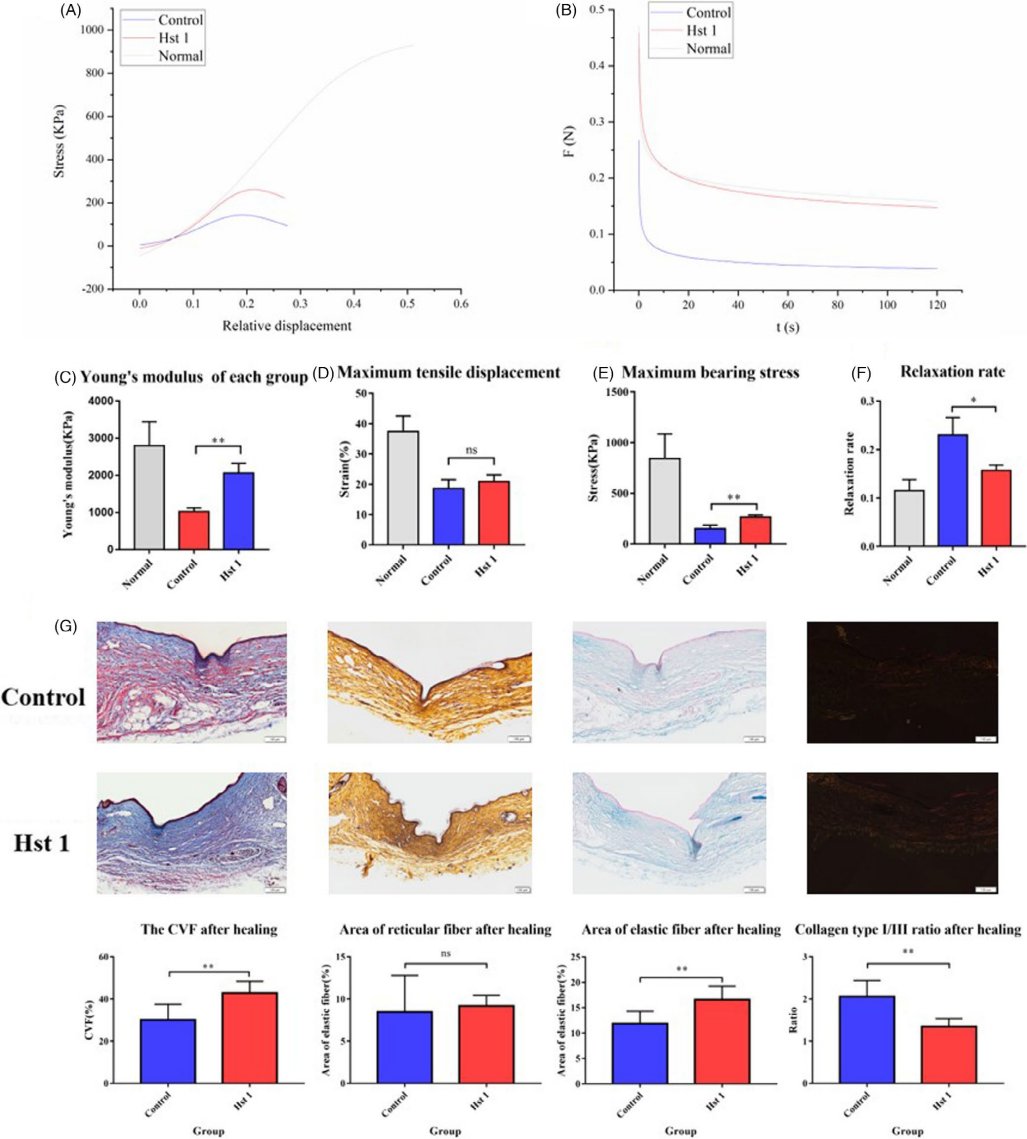
Detection of skin mechanical properties and fibre deposition 10 d after healing. A, At 10 d after healing, the skin on the wound of each group was taken for tensile fracture test, and the experimental results were recorded. Origin software was used to analyse the statistics. B, The Young's modulus of normal, control and Hst 1 group. C, The maximum tensile stress suffered by different groups. D, The maximum tensile length suffered by different groups. E, The stress relaxation was tested on the wound skin of the two groups 10 d after healing, and data were analysed by the Origin software. F, The relaxation rate of different groups. G, The staining of collagen fibre, reticular fibre and elastic fibre on the wound 10 d after healing and the distribution of type I collagen and type III collagen was staining by Sirius red staining. The data provided are one of the representative data. All experiments were repeated three times in an independent occasion. Data are shown as mean ± SE. (*P < .05; **P < .01)

The mechanical properties of the skin were detected 7 days after wound healing. Both tensile fracture and stress relaxation experiments showed that the mechanical properties of wound skin treated with Hst 1 were better than control group (Figure 4A‐F). The wound skin treated with Hst 1 had more collagen, reticular and elastic fibres in the wound local skin after healing (Figure 4G). In the healed skin, the proportion of type III collagen in Hst 1 group increased (Figure 4G).

### 3.5 The biological properties and functions of fibroblasts were regulated by Hst 1 in vitro

**FIGURE 5:**
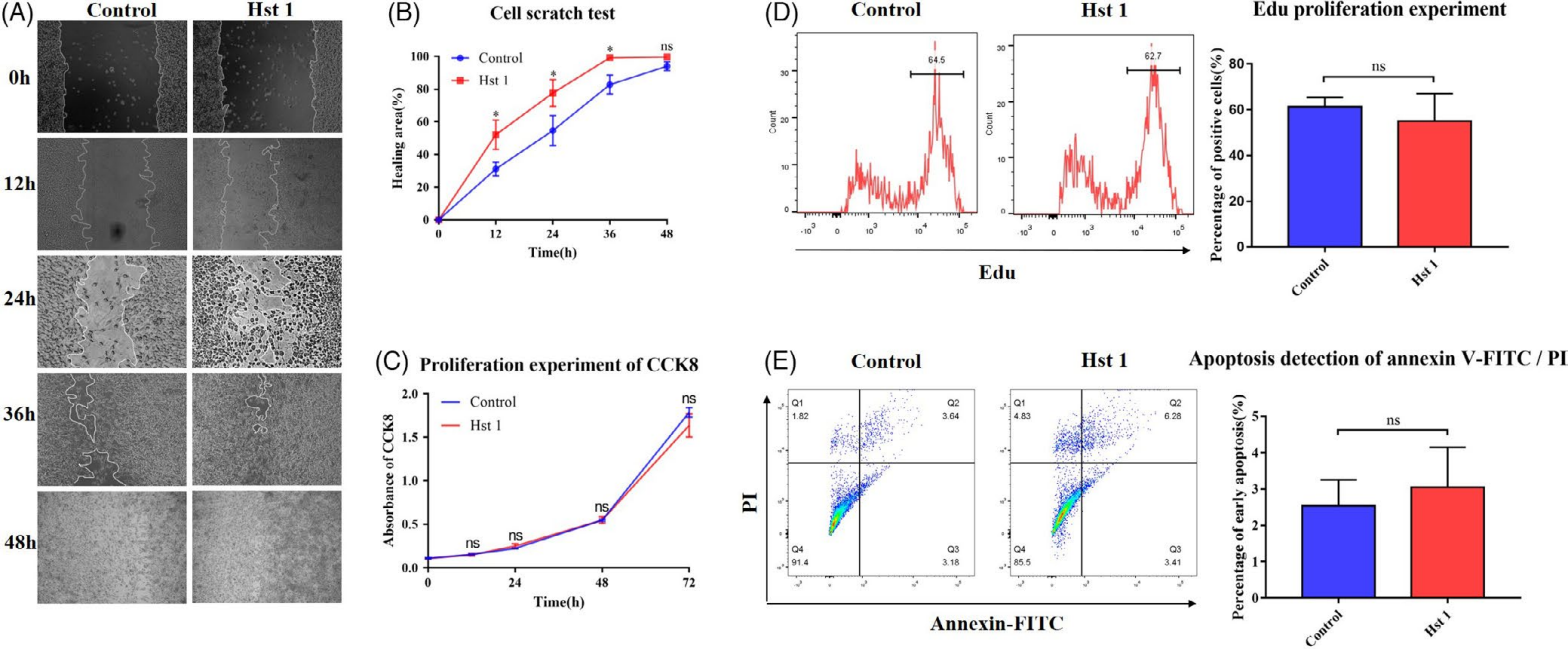
Effect of Hst 1 on the characteristics of 3T3 cells. A, Migration experiment of 3T3 cells. The results of cell scratch test were photographed by light microscope. B, The area of uncured scratch was calculated by image analyser. C, Fibroblast viability test. CCK8 was used to test the viability in different groups, and the results were detected by spectrophotometer. Vertical axis, absorbance; horizontal axis, time. D, Detection of cell proliferation ability by Edu test. The expression of Edu in 3T3 cells was detected after treated with Hst 1 for 24 h, and the result showed non‐statistical difference. E, Apoptosis was detected by flow cytometry with Annexin V‐FITC/PI staining. Q2 represented the late apoptotic cells, Q3 for the early apoptotic cells and Q4 for living cells. The control group was cultured with complete DEME medium (10% FBS), and Hst 1 group was cultured with complete DEME medium (10% FBS) including 10 μmol/L Hst 1. Data are shown as mean ± SE. (*P < .05; **P < .01)

**FIGURE 6:**
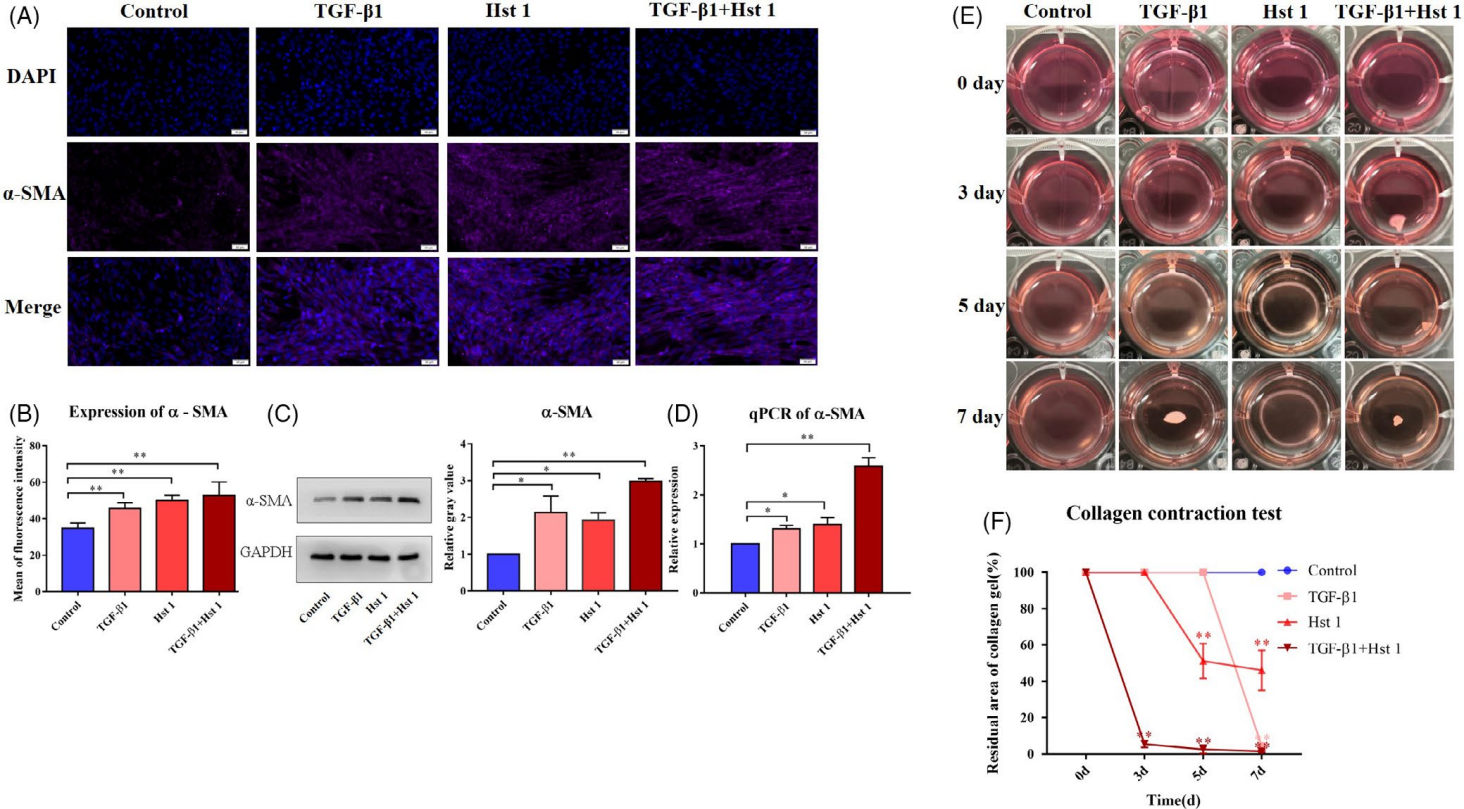
Transformation ability to myofibroblast and function of 3T3 cells. A‐D, The control group was cultured with DMEM medium (10% FBS), and the experimental group was added with 10 ng/mL TGF ‐β1, 10 μmol/L Hst 1 or both of them, respectively. Immunofluorescence staining (DAPI was labelled blue fluorescence and α‐SMA was purple), western blot and quantitative PCR were used to detect the expression of α‐SMA. E, Collagen contractive function of fibroblasts. Fibroblasts were added into the collagen of rat tail after gelatinization, and the factors were added to stimulate the cells as mentioned above. F, The contraction area of collagen was calculated by the image analyser. The data of each group were compared with the control group. The data provided are one of the representative data. All experiments were repeated three times in an independent occasion. Data are shown as mean ± SE. (*P < .05; **P < .01)

**FIGURE 7:**
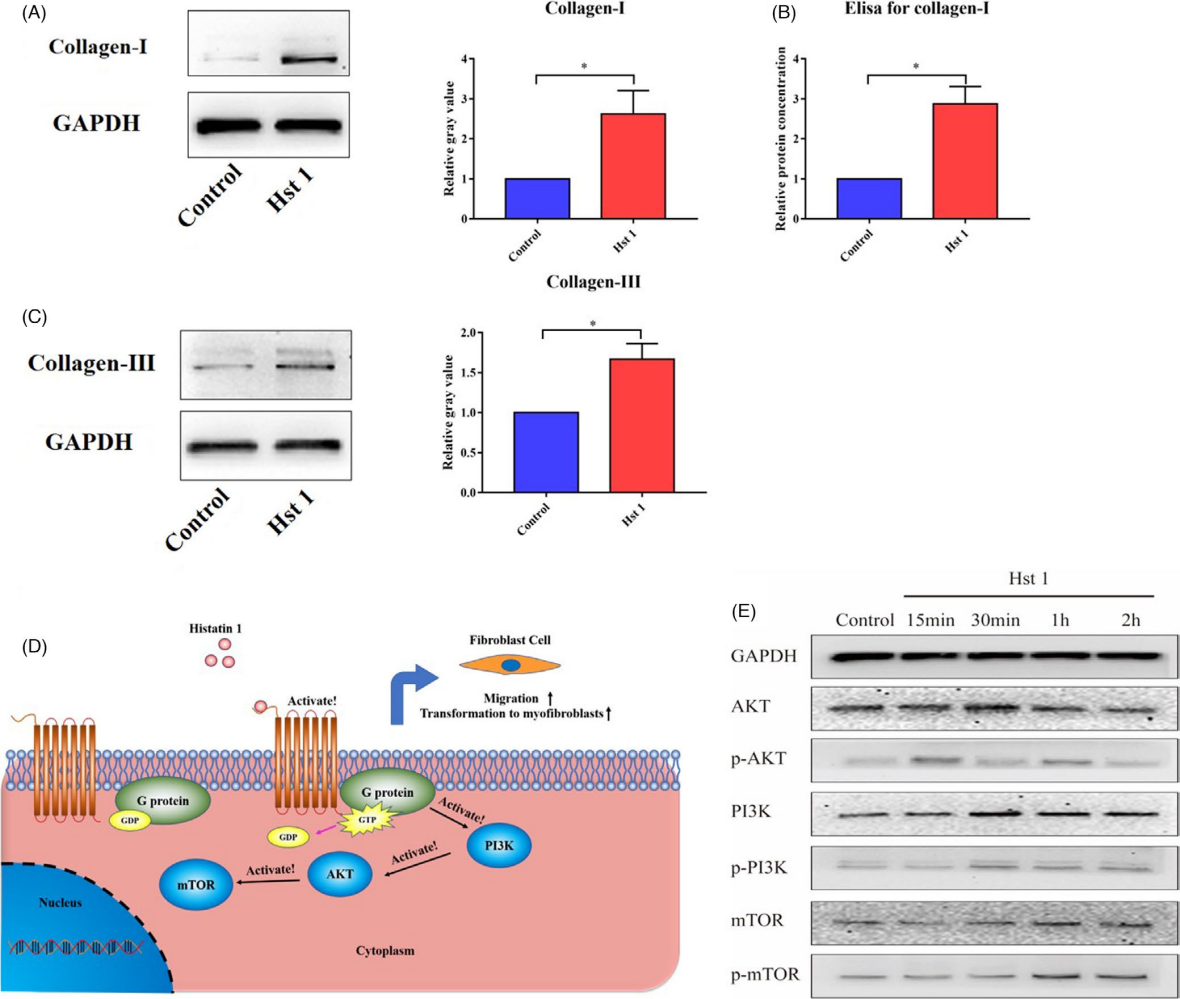
The collagen secretion function of 3T3 cells and the changes of mTOR pathway related signalling molecules after Hst 1 treatment. A, The secretion of type I collagen in 3T3 cells after treating with Hst 1 for 3 d, vertical axis, relative gray value; horizontal axis, group. B, After treatment with Hst 1 for 3 d, the secretion of extracellular type I collagen was detected by ELISA kit, vertical axis, relative protein concentration; horizontal axis, group. C, The secretion of type III collagen in 3T3 cells after treating with Hst 1 for 3 d, vertical axis, relative gray value; horizontal axis, group. D, The possible mechanism of Hst 1 regulating fibroblast function by activating mTOR signalling pathway. E, Hst 1 was used to stimulate the cells in vitro, and the cell proteins were extracted at 15 min, 30 min, 1 h and 2 h, respectively. The expression of AKT, PI3K, mTOR and their phosphorylated proteins was detected by Western blot. Data are shown as mean ± SE. (*P < .05; **P < .01)

Scratch test results showed that the migration ability of fibroblasts was enhanced and scratch healing speed was accelerated after Hst 1 treatment (Figure 5A,B). In addition, Hst 1 had no significant effect on the vitality, proliferation and apoptosis of fibroblasts (Figure 5C‐E). Fluorescence staining showed that Hst 1 could promote the transformation of fibroblasts similar to TGF‐β1, could increase the expression of α‐SMA in fibroblasts and transform them into myofibroblasts (Figure 6A,B). Western blot and qPCR results showed consistent results with immunofluorescence staining at protein and mRNA levels, respectively (Figure 6C,D). The results of cell contraction test showed that Hst 1, like TGF‐β1, could enhance the contraction function of fibroblasts (Figure 6E,F). In addition, the functions of fibroblasts to secrete type Ⅰ collagen and type III collagen were improved (Figure 7A‐C).

### 3.6 In fibroblasts, Hst 1 could activate mTOR signalling pathway related signalling molecules

The results showed that the phosphorylation levels of Akt, PI3K and mTOR were increased in fibroblasts stimulated by Hst 1 in vitro(Figure 7D,E).

## 4 DISCUSSION

Histatin 1 has been widely reported to promote acute wound healing in previous researches.^21^ However, most of the researches only reported the phenomena in vivo, including the promotion of wound healing speed, vascularization and inhibition of inflammatory reaction induced by Hst 1, while several of them studied the regulatory effect of Hst 1 on epidermal cells and vascular endothelial cells and proposed the possible mechanism. So far, there is no systematic study on the effect of Hst1 on fibroblast production. In this study, we confirmed that Hst 1 can accelerate the speed of wound healing through fibroblast contraction, collagen deposition and improve the mechanical properties after healing. These effects are achieved through Hst 1 promoting the migration of fibroblasts and their transformation into myofibroblasts.

The repair of the missing tissue in the wound is completed by contraction and reepithelization in mice. In addition, contraction was dominant, completing about 88% of wound closure.^22^ In wound healing, fibroblasts contact with the surrounding tissue and stretch to make the surrounding tissue fill the wound defect.^23^ Our experimental results showed that in animal experiments, the speed of wound reduction is obviously accelerated after treating by Hst 1. Previous studies generally believed that this result was due to promotion of epidermal cells migration and acceleration of the re‐epithelialization process. However, our study found that Hst 1 can promote the migration of fibroblasts, so that fibroblasts can gather in the wound and transform into myofibroblasts to enhance the contraction ability. Collagen contraction test in vitro also proved that Hst 1 could enhance the contraction function of fibroblasts. In collagen contraction test, although the contraction amplitude of Hst 1 group was smaller than that of TGF‐β1 group, it began to contract earlier and was significantly better than that of control group, in which there was almost no contraction after culturing for a week. For the number of cells mixed in each group was the same and Hst 1 had no effect on the proliferation of fibroblasts, Hst 1 can enhance the contractile function of fibroblasts directly rather than by other indirect methods, such as increasing the number of fibroblasts. The contraction ability of fibroblasts is closely related to the expression of α‐SMA.^24^, ^25^ α‐SMA is mainly expressed when fibroblasts transform into myofibroblasts and regulated by TGF‐β1.^7^, ^10^, ^26^ As shown in the results, adding Hst 1 simply can also induce fibroblasts to transform into myofibroblasts and increase the expression of α‐SMA in vitro, which is similar to that of TGF‐β1.

In addition to contraction, collagen secretion is one of the important functions of fibroblasts. Previous studies have also proved that Hst 1 can promote the density of collagen in the wound local site.^18^ Myofibroblasts, which can express α‐SMA, transformation from fibroblasts are the main source of extra cellular matrix (ECM) proteins during wound healing.^27^, ^28^ In the process of wound healing, insufficient collagen production will seriously affect the formation of ECM and granulation tissue, thus delaying wound healing.^11^, ^29^, ^30^ Moreover, the deposition and regular arrangement of collagen can improve the tissue remodelling.^31^ Our study further confirmed that Hst 1 can promote ECM density and granulation tissue formation in the early stage of wound healing. In addition, we tested the mechanical properties of the wound skin after 10 days of healing. Tensile fracture and stress relaxation tests showed that the mechanical properties of wound skin treated with Hst 1 were better than control group. The mechanical properties of skin are affected by the size and number of collagen.^32^, ^33^

Previous studies have proved that Hst 1 can enter cells directly.^14^ However, for the subsequent molecular reaction has not been confirmed and Hst 1 will be degraded in a short time in the cell,^34^ it is also believed that Hst 1 can bind to cell surface receptors, such as G‐protein‐coupled receptors and activate intracellular signalling pathways, leading to a series of subsequent reactions.^35^ ILK‐PI3K/AKT signalling pathway can promote the transformation of fibroblasts into myofibroblasts, enhance their contractile function and promote wound healing.^22^, ^36^ Our study confirmed that Hst 1 can activate PI3K/AKT/mTOR signalling pathway and has a similar positive regulatory effect on fibroblasts. In addition, activation of PI3K/AKT signalling pathway can enhance cell migration ability.^37^, ^38^ The tuberous sclerosis protein complex is phosphorylated by AKT, and the complex from GTPase Rheb is separated to activate mTOR.^39^ Related studies have shown that Akt regulates cell migration by activating mTOR.^40^, ^41^ Therefore, we consider that the activation and migration promoting effect of Hst 1 on fibroblasts is accomplished through the activation of PI3K/AKT/mTOR. Not only fibroblasts, but also Hst 1 has been proved to promote the migration of a variety of cells. In this study, we have proved that Hst 1 can activate PI3K/AKT/mTOR signalling pathway in fibroblasts, so we speculate that this activation not only exists in fibroblasts, but also is an important mechanism of Hst 1's extensive migration promoting effect.

In summary, we have made a comprehensive study on the regulation of Hst 1 on fibroblast function in wound healing in vivo and in vitro. In addition, the mechanism of its effect was explored in vitro. Hst 1 can promote the migration of fibroblasts and the transformation of fibroblasts into myofibroblasts, thus expressing stronger contractile and collagen secretion functions. In vivo, the wound healing rate was significantly accelerated and the mechanical properties of the healed skin were improved. This is related to the activation of mTOR signalling pathway by Hst 1 in fibroblasts, but the more detailed mechanism remains to be further studied.

## CONFLICT OF INTEREST

Authors have declared that they have no conflicts of interest to this study.

## AUTHOR CONTRIBUTIONS

The author's contribution to the article is as follows: GW and XF designed the study; LC, XL, ZY, SP and CC performed the experiments in vivo; YK, LC, JW, XZ and XH performed the experiments in vitro; LC and XL collected the data; PX and YD analysed the data; LC and FT wrote the manuscript; GW and XF supervised this study. All authors read and approved the final manuscript.

## Data Availability

All data generated or analysed during this study are included in the published article and its supplementary information files.
